# Simultaneous Sensor and Actuator Fault Reconstruction by Using a Sliding Mode Observer, Fuzzy Stability Analysis, and a Nonlinear Optimization Tool

**DOI:** 10.3390/s22186866

**Published:** 2022-09-10

**Authors:** Samira Asadi, Mehrdad Moallem, G. Gary Wang

**Affiliations:** School of Mechatronic Systems Engineering, Simon Fraser University, Surrey, BC V3T 0A3, Canada

**Keywords:** actuator and sensor faults, TS fuzzy system, sliding mode observer (SMO), *H*_∞_ performance, non-quadratic Lyapunov function (NQLF), fmincon, fault reconstruction

## Abstract

This paper proposes a Takagi–Sugeno (TS) fuzzy sliding mode observer (SMO) for simultaneous actuator and sensor fault reconstruction in a class of nonlinear systems subjected to unknown disturbances. First, the nonlinear system is represented by a TS fuzzy model with immeasurable premise variables. By filtering the output of the TS fuzzy model, an augmented system whose actuator fault is a combination of the original actuator and sensor faults is constructed. An $H_{\infty }$ performance criteria is considered to minimize the effect of the disturbance on the state estimations. Then, by using two further transformation matrices, a non-quadratic Lyapunov function (NQLF), and fmincon in MATLAB as a nonlinear optimization tool, the gains of the SMO are designed through the stability analysis of the observer. The main advantages of the proposed approach in comparison to the existing methods are using nonlinear optimization tools instead of linear matrix inequalities (LMIs), utilizing NQLF instead of simple quadratic Lyapunov functions (QLF), choosing SMO as the observer, which is robust to the uncertainties, and assuming that the premise variables are immeasurable. Finally, a practical continuous stirred tank reactor (CSTR) is considered as a nonlinear dynamic, and the numerical simulation results illustrate the superiority of the proposed approach compared to the existing methods.

## 1. Introduction

Over the past few decades, the reliability and safety of industrial systems has attracted considerable attention. As a consequence, fault-tolerant control (FTC) has received considerable attention in different fields [1,2]. There are different classifications for FTCs. In general, FTCs are classified into passive and active classifications. Active fault-tolerant controllers compensate for the effects of the occurred faults by using early information obtained from fault detection and isolation (FDI) schemes, which leads to a more flexible dynamic [3]. Consequently, FDI is becoming an attractive topic in different research fields. Observer-based methods are one of the most popular model-based FDIs. The main idea of observer-based FDIs is to construct a residual based on the measured output of the systems or to reconstruct the fault directly. Sliding mode observer (SMO) works based on the second approach, which detects the faults while determining the dynamic behavior [4,5]. SMOs are more insensitive to the unknown uncertainties occurring in the system compared to other observers like unknown input observers (UIOs) [6].

First, SMO observers were developed for linear dynamic systems; however, most actual physical systems are often nonlinear. Currently, lots of SMO-based fault reconstruction methods have been developed for uncertain nonlinear systems. In ref. [7], by considering a filter of the measured output vector, the original system with sensor and actuator faults is transformed into an augmented system with just the actuator fault and unknown inputs. Nevertheless, the classes of nonlinear systems considered in most of the papers are limited and cannot represent a general model for real systems [8,9].

Takagi–Sugeno (TS) fuzzy models can represent the behavior of nonlinear systems while keeping the simplicity of the linear models. A TS fuzzy representation is a convex nonlinear aggregation of several linear systems. Because the parameters of a TS fuzzy representation satisfy the convex sum, it is interesting to investigate the properties of the TS system based on its local linear vertices. With the advent of TS fuzzy systems, TS-based FDI techniques emerged to tackle a broader range of nonlinear systems [10]. By changing a nonlinear system to a TS system, some local linear systems are created, representing the behavior of the nonlinear system in a specific operating area. These local linear systems can be aggregated by using an interpolation mechanism. Thus, TS fuzzy models can represent the actual nonlinear behavior while maintaining the simplicity of linear models. Thus, an efficient FDI can be obtained by combining the SMO, which is robust to the uncertainties, and the TS fuzzy model, which causes simplicity in the design process. Recently, several researchers have utilized TS-based SMOs for fault detection and isolation in continuous-time and discrete-time systems [11,12]. However, in the methods developed in these articles, it is assumed that the premise variables are measurable, which reduces the applicability of these approaches. To deal with this problem, an FDI approach for stability analysis of the TS fuzzy systems with immeasurable premise variables was proposed in [13,14].

In [15], simultaneous actuator and sensor faults in a nonlinear system represented by a TS fuzzy model are reconstructed by using an SMO and considering $H_{\infty }$ performance criteria to reduce the effect of disturbance, whereas [16] does the same procedure for the fault reconstructions and both of the exogenous disturbance and the system faults are reconstructed. However, in refs. [15,16] quadratic Lyapunov functions (QLFs) are used to design the observers. By using the QLF for TS fuzzy systems with a large number of fuzzy rules can cause undesired performance or unfeasible solutions. Consequently, refs. [17,18] offered to use a non-quadratic Lyapunov function (NQLF) to design the TS-based SMO for the FDI purposes. In all these papers, a linear optimization approach based on linear matrix inequalities (LMIs) is utilized, making the stability analysis more complex and using some approximations and lemmas to prove the stability conditions.

In this paper, a TS fuzzy-based SMO with immeasurable premise variables is designed to reconstruct simultaneous actuator and sensor faults in a nonlinear system exposed to an unknown disturbance. Then, the states and faults are estimated. The stability of the proposed observer is guaranteed by using the NQLF and fmincon as a nonlinear optimization tool in MATLAB. In addition, $H_{\infty }$ performance criteria are considered to minimize the effect of disturbances and uncertainties on the estimation error and the fault estimations. By using the NQLF, a generalized eigenvalue problem is proposed, which maximizes the admissible Lipschitz constant and minimizes the disturbance effects on the estimation error through a nonlinear optimization problem.

The main advantages of the proposed approach over the existing methods can be summarized as follows:

- Using nonlinear optimization tools instead of LMIs, which results in better accuracy.
- Utilizing NQLF, which leads to less conservative optimization conditions than simple quadratic Lyapunov functions.
- Assuming that the premise variables are immeasurable, which makes the proposed method applicable to a broader class of TS fuzzy systems.

This paper is organized as follows. Section 2 presents a TS fuzzy model with simultaneous actuator and sensor faults and disturbance and how to construct a fictitious system with just an actuator fault. In Section 3, the main results of this paper, including the sliding mode observer design and the sufficient conditions of stability of the estimation errors, are proposed and guarantee the $H_{\infty }$ performance simultaneously. Section 4 discusses the procedure of the actuator and sensor fault reconstructions. In Section 5, simulation results are given, and comparisons are discussed. Finally, in Section 6, the concluding remarks are given.

## 2. Preliminaries

Assume that a continuous-time nonlinear system affected by actuator and sensor faults and disturbance is given as

(1) $$\{\begin{array}{c} \begin{array}{c} \dot{x}\left(t\right)=f\left(x\left(t\right),u\left(t\right),f_{a}\left(t\right),d\left(x\left(t\right),u\left(t\right),t\right)\right)\; \end{array}\\ y\left(t\right)=Cx\left(t\right)+Nf_{s}\;\left(t\right)\;\;\;\;\;\;\;\;\;\;\;\;\;\;\;\;\;\;\;\;\;\;\;\;\;\;\;\;\;\;\;\;\;\;\;\;\;\;\; \end{array},$$

where $x\left(t\right)\in R^{n}$, $u\left(t\right)\in R^{m}$, $y\left(t\right)\in R^{p}$, $f_{a}\left(t\right)\in R^{q}$, $f_{s}\left(t\right)\in R^{h}$ and $d\left(x\left(t\right),u\left(t\right),t\right)\in R^{l}$ are the state, input, output, unknown actuator, and sensor faults, and the system uncertainty vectors, respectively. f and g are nonlinear smooth functions. By using sector nonlinearity transformation, the nonlinear model (1) can be replaced by the following TS fuzzy model

(2) $$\{\begin{array}{l} \begin{array}{c} \dot{x}\left(t\right)=\overset{r}{\underset{i=1}{\sum }}\mu_{i}\left(\xi \left(t\right)\right)\left\{\begin{array}{c} A_{i}x\left(t\right)+B_{i}u\left(t\right)+M_{i}f_{a}\left(t\right)\\ +D_{i}d\left(x\left(t\right),u\left(t\right),t\right) \end{array}\right\}\; \end{array}\\ y\left(t\right)=Cx\left(t\right)+Nf_{s}\left(t\right)\;\;\;\;\;\;\;\;\;\;\;\;\;\;\;\;\;\;\;\;\;\;\;\;\;\;\;\;\;\;\;\;\;\;\;\;\;\;\;\;\;\;\;\;\;\;\;\; \end{array},$$

Where C and N are known full rank matrices with appropriate dimensions. $A_{i}$, $B_{i}$, $M_{i}$, and $D_{i}$ are real known matrices, r represents the number of fuzzy rules and $\mu_{i}\left(\xi \left(t\right)\right)$ are the fuzzy membership functions depending on the unmeasurable variable vector ξ(t) and satisfy the following so-called convex sum property

(3) $$\{\begin{array}{l} 0\leq \mu_{i}\left(\xi \left(t\right)\right)\leq 1\\ \overset{r}{\underset{i=1}{\sum }}\mu_{i}\left(\xi \left(t\right)\right)=1 \end{array}.$$

In the rest of the paper, (t) is dropped from the equations, d, $\mu_{i}$ and ${\hat{\mu }}_{i}$ denote d(x,u,t), $\mu_{i}\left(\xi \left(t\right)\right)$, and $\mu_{i}\left(\hat{\xi }\left(t\right)\right)$ and the mark (*) denotes the transposed element in a symmetric matrix.

To build a system with just an actuator fault and then use the actuator fault reconstruction concepts, the output is passed through an orthogonal matrix $T_{r}\in R^{p\times p}\;$ and an augmented TS system of order n+h can be obtained as

(4) $$\{\begin{array}{l} \dot{X}=\overset{r}{\underset{i=1}{\sum }}\mu_{i}\left\{A_{i}X+{ℬ}_{i}u+D_{i}d+{ℳ}_{i}f_{a}+Nf_{s}\right\}\\ Y=CX\;\;\;\;\;\;\;\;\;\;\;\;\;\;\;\;\;\;\;\;\;\;\;\;\;\;\;\;\;\;\;\;\;\;\;\;\;\;\;\;\;\;\;\;\;\;\;\;\;\;\;\;\;\;\;\;\;\;\;\;\;\;\;\;\;\;\;\;\;\;\; \end{array},$$

where $X={\left[x^{T}\;z^{T}\right]}^{T}\in R^{n+h}$, $Y={\left[y_{1}^{T}\;z^{T}\right]}^{T}\in R^{p}$, and

(5) $$A_{i}=\left[\begin{array}{cc} A_{i} & 0\\ A_{f}C_{2} & -A_{f} \end{array}\right],{ℬ}_{i}=\left[\begin{array}{c} B_{i}\\ 0 \end{array}\right],D_{i}=\left[\begin{array}{c} D_{i}\\ 0 \end{array}\right],\;\;{ℳ}_{i}=\left[\begin{array}{c} M_{i}\\ 0 \end{array}\right],N=\left[\begin{array}{c} 0\\ A_{f}N_{2} \end{array}\right],\;C=\left[\begin{array}{cc} C_{1} & 0\\ 0 & I_{h} \end{array}\right].$$

$-A_{f}\in R^{h\times h}$ is an arbitrary stable matrix, $z\in R^{h}$ and $N_{2}\in R^{h\times h}$. $T_{r}$ can be obtained by QR reduction of the matrix N.

By defining

(6) $$\phi :=\sum_{i=1}^{r}\left(\mu_{i}-{\hat{\mu }}_{i}\right)\left\{A_{i}X++{ℬ}_{i}u+D_{i}d+{ℳ}_{i}f_{a}+Nf_{s}\right\},$$

where $\hat{x}$ is the estimation of the x, the TS system (4) can be derived as

(7) $$\{\begin{array}{c} \begin{array}{c} \dot{X}=\overset{r}{\underset{i=1}{\sum }}{\hat{\mu }}_{i}\left\{A_{i}X+{ℬ}_{i}u+D_{i}d+{ℳ}_{i}f_{a}+Nf_{s}+\phi \right\}\;\; \end{array}\\ \begin{array}{c} Y=CX\;\;\;\;\;\;\;\;\;\;\;\;\;\;\;\;\;\;\;\;\;\;\;\;\;\;\;\;\;\;\;\;\;\;\;\;\;\;\;\;\;\;\;\;\;\;\;\;\;\;\;\;\;\;\;\;\;\;\;\;\;\;\;\;\;\;\;\;\;\;\;\;\;\;\;\;\;\;\;\;\; \end{array} \end{array}.$$

Moreover, the nonlinear term ϕ is assumed to satisfy the Lipschitz condition as

(8) $$\phi \leq \gamma ∥x-\hat{x}∥.\;\;\;\;\;\;\;\;\;\;\;\;\;\;\forall x,\hat{x}\in R^{n}.$$

To design a sliding mode observer, some assumptions and lemmas are needed as follows.

**Assumption** **1.**
(9) $$rank\left(C\left[{ℳ}_{i}\;N\right]\right)=q+h$$

**Assumption** **2.**
(10) n>p≥q+h

**Assumption** **3.**
(11) $$rank\left[\begin{array}{ccc} sI_{n+h-}A_{i} & {ℳ}_{i} & N\\ C & 0 & 0 \end{array}\right]=n+2h+q$$

*for all* s *satisfying* Re(s)≥0 *holds.*

**Lemma** **1.**

(a) *If Assumptions 1 and 2 are satisfied, then there exist changes of coordinates* ${\bar{T}}_{i}$ *such that*
(12) $$A_{i}=\left[\begin{array}{cc} A_{11.i} & A_{12.i}\\ \left[\begin{array}{c} A_{211.i}\\ A_{212.i} \end{array}\right] & A_{22.i} \end{array}\right],\;{ℳ}_{i}=\left[\begin{array}{c} 0\\ {ℳ}_{2.i} \end{array}\right],\;N=\left[\begin{array}{c} 0\\ N_{2} \end{array}\right],\;D_{i}=\left[\begin{array}{c} D_{1.i}\\ D_{2.i} \end{array}\right],\;C=\left[0\;\;T_{0}\right],$$
  *where* $A_{11.i}\in R^{\left(n+h-p\right)\times \left(n+h-p\right)}$ *,* $A_{211.i}\in R^{\left(p-q-h\right)\times \left(n+h-p\right)}$ *,* $D_{2.i}\in R^{p\times l}$ *, and* $T_{0}\in R^{p\times p}$ *is an orthogonal matrix. Matrices* ${ℳ}_{2.i}\in R^{p\times q}$ *,* $N_{2}\in R^{p\times h}\;$ *can have the following structure:*
(13) $${ℳ}_{2.i\;}=\left[\begin{array}{c} 0\\ {ℳ}_{0.i} \end{array}\right],\;N_{2}=\left[\begin{array}{c} 0\\ N_{0} \end{array}\right].$$
*With* ${ℳ}_{0.i}\in R^{\left(q+h\right)\times q}$ *,* $N_{0}\in R^{\left(q+h\right)\times h}$ *are nonsingular.*
(b) *The pairs (* $A_{11.i},A_{21.i}$ *) are detectable if and only if the invariant zeros of {* $A_{i},\;[{ℳ}_{i}\;N],C$ *} lie in* ${\mathbb{C}}^{-}$ *and it happens if and only if Assumption 3 is satisfied.*

**Assumption** **4.** *The unknown vectors* $f_{a}$ *and* $f_{s}\;$ *and the derivatives of the* $\mu_{i}$ *for* i∈{1.….r} *are assumed to be norm bounded by some known constants. Therefore,*

(14) $$∥f_{a}∥\leq \rho_{a};\;\;\;∥f_{s}∥\leq \rho_{s};\;\;\;∥{\dot{\mu }}_{i}∥\leq \rho_{mi}.$$

**Lemma** **2.** *Ref.* [19] *parameterized linear matrix inequality (PLMI)* $\overset{r}{\underset{i=1}{\sum }}\overset{r}{\underset{j=1}{\sum }}\mu_{i}\mu_{j}Q_{ij}<0$ *is fulfilled* *if the following conditions hold:*

(15) $$\{\begin{array}{c} R_{ii}<0\;\;\;\;\;\;\;\;\;\;\;\;\;\;\;\;\;\;\;\;\;\;\;\;\;\;\;\;\;\;\;\;\;\;\;\;\;\;\;\;\;for\;i=1,\ldots ,r\;\;\;\;\;\;\;\;\;\;\\ \frac{2}{r-1}R_{ii}+R_{ij}+R_{ji}<0\;\;\;\;\;\;\;\;\;for\;i\neq j=1,\ldots ,r\; \end{array}.$$

## 3. TS Fuzzy-Based Sliding Mode Observer Design

The proposed TS sliding mode observer for the nonlinear system (2) in the new coordinate (10) is as follows:

(16) $$\left\{\begin{array}{c} \dot{\hat{X}}=\sum_{i=1}^{r}{\hat{\mu }}_{i}\left\{A_{i}\hat{X}+B_{i}u+G_{l.i}e_{Y}+G_{n.i}v_{a.i}+G_{n.i}v_{s}\right\}\\ \hat{Y}=C\hat{X} \end{array}\right.$$

where $G_{n.i}$ and $G_{l.i}$ are design matrices of the observer that will be derived through Theorem 1. $e_{Y}:=Y-\hat{Y}$ represents the output error estimation, $\nu_{a.i}\;$ and $\nu_{s}$ are the equivalent output error injections that are used to compensate the errors due to the actuator fault and sensor fault, respectively, and have the following structure:

(17) $$\nu_{a,i}=\{\begin{array}{c} \eta_{a,i}\frac{∥e_{Y}∥}{e_{Y}}\;\;\;\;\;e_{Y}\neq 0\;\;\;\\ 0\;\;\;\;\;\;\;\;\;\;\;\;\;\;\;otherwise \end{array}\;\nu_{s}=\{\begin{array}{c} \eta_{s}\frac{∥e_{Y}∥}{e_{Y}}\;\;\;\;\;\;\;e_{Y}\neq 0\;\;\;\\ 0\;\;\;\;\;\;\;\;\;\;\;\;\;\;\;otherwise \end{array},$$

where $\eta_{a.i}$ and $\eta_{s}$ are two positive scalars such that

(18) $$\begin{array}{c} \eta_{a.i}\geq \rho_{a}∥T_{0}{ℳ}_{2.i}∥\underset{j}{\max}(\frac{∥P_{2.j}∥}{\lambda_{min}\left(P_{2.j}\right)})+w_{a.i}\;\;\\ \eta_{s}\geq \rho_{s}∥T_{0}N_{2}∥\underset{j}{\max}(\frac{P_{2.j}}{\lambda_{min}\left(P_{2.j}\right)})+w_{s}\;\;\;\;\;\;\;\;\;\; \end{array}\;\forall i,j\in \left\{1.\ldots .r\right\}.$$

$w_{a.i}$ and $w_{s}$ are two arbitrary positive constants.

The observer (16) guarantees that the state estimation error converges to a pre-designed sliding surface in finite time and then, asymptotically to zero. Define state estimation error as $e:=X-\hat{X}$. By subtracting the observer dynamics from the system dynamic (7) in the new coordinate (12), the state estimation error dynamic can be given as

(19) $$\begin{array}{c} \dot{e}=\overset{r}{\underset{i=1}{\sum }}{\hat{\mu }}_{i}\left\{\begin{array}{c} \left(A_{i}-G_{l.i}\;C\right)e+{ℳ}_{i}f_{a}-G_{n.i}\nu_{a.i}\\ +Nf_{s}-G_{n.i}\nu_{s}+D_{i}d+\phi \end{array}\right\} \end{array}.$$

By partitioning ϕ as $\phi ={\left[\phi_{1}^{T}\;\phi_{2}^{T}\right]}^{T}$ and applying a further change of coordinates

(20) $$\begin{array}{c} T_{L.i}=\left[\begin{array}{cc} I_{n+h-p} & L_{i}\\ 0 & T_{0} \end{array}\right],\;L_{i}=\left[{\bar{L}}_{i}\;\;\;\;\;0\right]\in R^{\left(n+h-p\right)\times p}\;\; \end{array}$$

where ${\bar{L}}_{i}\in R^{\left(n+h-p\right)\times \left(p-q-h\right)}$ is a stabilizing gain matrix, it is straightforward to see that

(21) $$\left\{\begin{array}{c} {\tilde{A}}_{i}=\left[\begin{array}{cc} A_{11.i}+L_{i}A_{21.i} & {\tilde{A}}_{12.i}\\ T_{0}A_{21.i} & {\tilde{A}}_{22.i} \end{array}\right]\\ {\tilde{M}}_{i}=\left[\begin{array}{c} 0\\ T_{0}M_{2.i} \end{array}\right]\\ \tilde{N}=\left[\begin{array}{c} 0\\ T_{0}N_{2} \end{array}\right]\\ {\tilde{D}}_{i}=\left[\begin{array}{c} D_{1.i}+L_{i}D_{2.i}\\ T_{0}D_{2.i} \end{array}\right]\\ \tilde{C}=\left[\begin{array}{cc} 0 & I_{P} \end{array}\right]\\ {\tilde{G}}_{n,i}=\left[\begin{array}{c} 0\\ I_{p} \end{array}\right]\\ {\tilde{G}}_{l,i}=\left[\begin{array}{c} {\tilde{A}}_{12,i}\\ {\tilde{A}}_{22.i}-A_{s.i} \end{array}\right]\\ \tilde{\phi }=\left[\begin{array}{c} T_{L.i}\phi_{1}\\ T_{L.i}\phi_{2} \end{array}\right] \end{array},\right.$$

where $A_{s.i}$ are arbitrary stable design matrices. Through the new coordinate, the error dynamic (19) can be re-written as

(22) $$\begin{array}{c} \dot{\tilde{e}}=\left[\begin{array}{c} {\dot{e}}_{1}\\ {\dot{e}}_{Y} \end{array}\right]=\overset{r}{\underset{i=1}{\sum }}{\hat{\mu }}_{i}\left\{\begin{array}{c} {\tilde{A}}_{t.\;i}\tilde{e}+T_{L.i}\phi +{\tilde{ℳ}}_{i}f_{a}-\\ {\tilde{G}}_{n.i}\nu_{a.i}+\tilde{N}f_{s}-{\tilde{G}}_{n.i}\nu_{s}+{\tilde{D}}_{i}d \end{array}\right\}\;\; \end{array}$$

where

(23) $$\begin{array}{c} {\tilde{A}}_{t,\;i}=\left[\begin{array}{cc} A_{11.i}+L_{i}A_{21.i} & 0\\ T_{0}A_{21.i} & A_{s.i}\; \end{array}\right]\;\; \end{array}.$$

The goal is to design the matrices $L_{i}$ such that the asymptotic stability of (22) is assured while the following specified $H_{\infty }$ performance is guaranteed:

(24) $$\begin{array}{c} ∥{\tilde{e}}^{2}∥\leq \vartheta^{2}∥d^{2}∥ \end{array}.$$

The following theorem provides sufficient conditions to ensure asymptotic stability of the state estimation error (22) with maximized admissible Lipschitz constant γ in (8) and minimized $H_{\infty }$ performance gain ϑ in (24).

**Theorem** **1.** *If there exist feasible solutions for the following optimization problem with a fixed scalar* 0≤λ≤1

(25) $$\begin{array}{cc} \min[\lambda (\sigma +\varepsilon )+(1-\lambda )\theta ] & \\ Subject\;to & for\;i=1,\ldots ,r\\ eig\left(R_{ii}\right)<0 & for\;i\neq j=1,\ldots ,r\\ eig\left(\frac{2}{r-1}R_{ii}+R_{ij}+R_{ji}\right) & for\;i=i=1,\ldots ,r\\ -eig\left(P_{1.i}\right)<0 & for\;i=i=1,\ldots ,r\\ -eig\left(P_{2.i}\right)<0 & \\ -\varepsilon <0 & \\ -\sigma <0 & \\ -\theta <0 \end{array}$$

*where*

(26) $$R_{ij}=\left[\begin{array}{ccc} \Phi_{1,ij} & {\left(P_{2,j}T_{0}A_{21,i}\right)}^{T} & \Phi_{3,ij}\\ P_{2,j}T_{0}A_{21,i} & \Phi_{2,ij} & P_{2,j}T_{0}D_{2,i}\\ \Phi_{3,ij}{}^{T} & {\left(P_{2,j}T_{0}D_{2,i}\right)}^{T} & -\beta I_{l} \end{array}\right]\;\Phi_{1.ij}={\left(A_{11.i}+L_{i}A_{21.i}\right)}^{T}P_{1j}+P_{1j}\left(A_{11.i}+L_{i}A_{21i}\right)+\varepsilon^{-1}P_{1.j}P_{1.j}+\left(\sigma^{-1}+1\right)I_{n+h-p}+\overset{r}{\underset{k=1}{\sum }}q_{mk}P_{1.k}\;\Phi_{2ij}=A_{s.i}^{T}P_{2.j}+P_{2.j}A_{s.i}+\varepsilon^{-1}P_{2.j}P_{2.j}+\left(\sigma^{-1}+1\right)I_{p}+\overset{r}{\underset{k=1}{\sum }}q_{mk}P_{2.k}\;\Phi_{3.ij}=P_{1.j}D_{1.i}+P_{1.j}L_{i}D_{2.i}$$

*and*eig*represents eigenvalues of a matrix, then, the estimation error (22) is asymptotically stable with the maximized admissible Lipschitz constant*$\gamma^{*}=\max\left(\gamma \right)=\frac{1}{∥T_{L}∥∥T_{L}{}^{-1}∥\sqrt{\varepsilon \sigma }}$ and the derived $L_{i}$ *matrices can be used for the purpose of simultaneous fault reconstruction.*

**Proof.**  The proof of this theorem is done by using a positive NQLF as follows

(27) $$\begin{array}{c} V={\tilde{e}}^{T}\left(\overset{r}{\underset{j=1}{\sum }}{\hat{\mu }}_{j}P_{j}\right)\tilde{e} \end{array},$$

where $P_{j}=\mathrm{diag}\left(P_{1j},P_{2j}\right)$ with $P_{1j}\in R^{\left(n+h-p\right)\times \left(n+h-p\right)}$ and $P_{2j}\in R^{p\times p}$ are symmetric positive definite matrices. The time derivative of the candidate Lyapunov function along the trajectory (22) is given by

(28) $$\dot{V}=\overset{r}{\underset{i=1}{\sum }}\overset{r}{\underset{j=1}{\sum }}{\hat{\mu }}_{i}{\hat{\mu }}_{j}\left\{{\tilde{e}}^{T}\left(A_{t.i}{}^{T}P_{j}+P_{j}A_{t.i}+\overset{r}{\underset{k=1}{\sum }}{\dot{\hat{\mu }}}_{k}P_{k}\right)\tilde{e}+2{\tilde{e}}^{T}P_{j}\left(T_{L.i}\phi +{\tilde{ℳ}}_{i}f_{a}-{\tilde{G}}_{n.i}\nu_{a.i}+\tilde{N}f_{s}-{\tilde{G}}_{n.i}\nu_{s}+{\tilde{D}}_{i}d\right)\right\}.$$

From (14), (17), (18) and (21), one has:

(29) $$\begin{array}{l} {\tilde{e}}^{T}P_{j}\left({\tilde{ℳ}}_{i}f_{a}-{\tilde{G}}_{n.i}\nu_{a.i}\right)\\ =e_{Y}{}^{T}P_{2.j}T_{0}{ℳ}_{2.i}f_{a}-\eta_{a.i}\frac{e_{Y}{}^{T}P_{2.j}e_{Y}}{∥e_{Y}∥}\\ \leq ∥e_{Y}{}^{T}P_{2.j}T_{0}{ℳ}_{2.i}f_{a}∥-\eta_{a.i}\frac{e_{Y}{}^{T}P_{2.j}e_{Y}}{∥e_{Y}∥}\\ \leq ∥e_{Y}{}^{T}P_{2.j}T_{0}{ℳ}_{2.i}f_{a}∥-\eta_{a.i}\lambda_{min}\left(P_{2.j}\right)∥e_{Y}∥\\ \leq ∥e_{Y}∥\left(\rho_{a}∥P_{2.j}∥∥T_{0}{ℳ}_{2.i}∥-\eta_{a.i}\lambda_{min}\left(P_{2.j}\right)\right)\\ \leq -w_{a.i}\lambda_{min}\left(P_{2.j}\right)∥e_{Y}∥\leq 0\\ {\tilde{e}}^{T}P_{j}\left(\tilde{N}f_{s}-{\tilde{G}}_{n.i}\nu_{s}\right)\\ =e_{Y}{}^{T}P_{2.j}T_{0}N_{2}f_{s}-\eta_{s}\frac{e_{Y}{}^{T}P_{2.j}e_{Y}}{∥e_{Y}∥}\\ \leq ∥e_{Y}{}^{T}P_{2.j}T_{0}N_{2}f_{s}∥-\eta_{s}\frac{e_{Y}{}^{T}P_{2.j}e_{Y}}{∥e_{Y}∥}\\ \leq ∥e_{Y}{}^{T}P_{2.j}T_{0}N_{2}f_{s}∥-\eta_{s}\lambda_{min}\left(P_{2.j}\right)∥e_{Y}∥\\ \leq ∥e_{Y}∥\left(\rho_{s}∥P_{2.j}∥∥T_{0}N_{2}∥-\eta_{s}\lambda_{min}\left(P_{2.j}\right)\right)\\ \leq -w_{s}\lambda_{min}\left(P_{2.j}\right)∥e_{Y}∥\leq 0. \end{array}$$

From (14), one has

(30) $$\overset{r}{\underset{k=1}{\sum }}{\dot{\hat{\mu }}}_{k}P_{k}\leq \overset{r}{\underset{k=1}{\sum }}\rho_{mi}P_{k}.$$

By considering the fact that $2{\mathbb{P}}^{T}\mathbb{Q}\leq \frac{1}{\;\varepsilon }{\mathbb{P}}^{T}\mathbb{P}+\varepsilon {\mathbb{Q}}^{T}\mathbb{Q}$ with ε>0 and using (8), one obtains

(31) $$2{\tilde{e}}^{T}P_{j}T_{L}\phi \leq \frac{1}{\;\varepsilon }{\tilde{e}}^{T}P_{j}P_{j}\tilde{e}+\varepsilon \phi^{T}T_{L}{}^{T}T_{L}\phi \leq \frac{1}{\;\varepsilon }{\tilde{e}}^{T}P_{j}P_{j}\tilde{e}+\varepsilon \alpha^{2}{\tilde{e}}^{2},$$

where $\alpha :=∥T_{L}∥∥T_{L}{}^{-1}∥\gamma$. By Substituting (29)–(31) into (28), one has

(32) $$\dot{V}\leq \overset{r}{\underset{i=1}{\sum }}\overset{r}{\underset{j=1}{\sum }}{\hat{\mu }}_{i}{\hat{\mu }}_{j}\left\{{\tilde{e}}^{T}\left(A_{t.i}{}^{T}P_{j}+P_{j}A_{t.i}+\frac{1}{\;\varepsilon }P_{j}P_{j}+\varepsilon \alpha^{2}I_{n+h}+\overset{r}{\underset{k=1}{\sum }}\rho_{mk}P_{k}\right)\tilde{e}+2{\tilde{e}}^{T}P_{j}{\tilde{D}}_{i}d\right\}.$$

By defining parameter $\sigma :={\left(\varepsilon \alpha^{2}\right)}^{-1}$ and the cost function as $J:=\dot{V}\left(\tilde{e}\right)+{\tilde{e}}^{T}\tilde{e}-\vartheta^{2}d^{T}d$, one has

(33) $$J\leq \overset{r}{\underset{i=1}{\sum }}\overset{r}{\underset{j=1}{\sum }}{\hat{\mu }}_{i}{\hat{\mu }}_{j}\left\{{\tilde{e}}^{T}\left(A_{t.i}{}^{T}P_{j}+P_{j}A_{t.i}+\varepsilon^{-1}P_{j}P_{j}+\sigma^{-1}I_{n+h}+I_{n+h}+\overset{r}{\underset{k=1}{\sum }}\rho_{mk}P_{k}\right)\tilde{e}+2{\tilde{e}}^{T}P_{j}{\tilde{D}}_{i}d-\beta d^{T}d\right\},$$

where $\beta :=\vartheta^{2}$. By placing (23)in (33) and considering the diagonal structure of $P_{j}$, the inequality (33) is continued as

(34) $$J\leq \overset{r}{\underset{i=1}{\sum }}\overset{r}{\underset{j=1}{\sum }}{\hat{\mu }}_{i}{\hat{\mu }}_{j}{\left[\begin{array}{c} e_{1}\\ e_{Y}\\ \xi \end{array}\right]}^{T}\Lambda \left[\begin{array}{c} e_{1}\\ e_{y}\\ \xi \end{array}\right]<0,$$

where

(35) $$\Lambda =\left[\begin{array}{ccc} \Phi_{1,ij} & {\left(P_{2,j}T_{0}A_{21,i}\right)}^{T} & \Phi_{3,ij}\\ P_{2,j}T_{0}A_{21,i} & \Phi_{2,ij} & P_{2,j}T_{0}D_{2,i}\\ \Phi_{3,ij}{}^{T} & {\left(P_{2,j}T_{0}D_{2,i}\right)}^{T} & -\beta I_{l} \end{array}\right]\;\Phi_{1.ij}={\left(A_{11.i}+L_{i}A_{21.i}\right)}^{T}P_{1.j}+P_{1j}\left(A_{11.i}+L_{i}A_{21.i}\right)+\varepsilon^{-1}P_{1.j}P_{1j}+\left(\sigma^{-1}+1\right)I_{n+h-p}+\overset{r}{\underset{k=1}{\sum }}q_{mk}P_{1.k}\;\Phi_{2.ij}=A_{s.i}^{T}P_{2.j}+P_{2.j}A_{s.i}+\varepsilon^{-1}P_{2.j}P_{2.j}+\left(\sigma^{-1}+1\right)I_{p}+\overset{r}{\underset{k=1}{\sum }}q_{mk}P_{2..k}\;\Phi_{3.ij}=P_{1.j}D_{1.i}+P_{1.j}L_{i}D_{2.i}.$$

Based on the Congruence [20], the inequality (35) is satisfied by

(36) $$\overset{r}{\underset{i=1}{\sum }}\overset{r}{\underset{j=1}{\sum }}{\hat{\mu }}_{i}{\hat{\mu }}_{j}\left[\begin{array}{ccc} \Phi_{1,ij} & {\left(P_{2,j}T_{0}A_{21,i}\right)}^{T} & \Phi_{3,ij}\\ P_{2,j}T_{0}A_{21,i} & \Phi_{2,ij} & P_{2,j}T_{0}D_{2,i}\\ \Phi_{3,ij}{}^{T} & {\left(P_{2,j}T_{0}D_{2,i}\right)}^{T} & -\beta I_{l} \end{array}\right]<0.$$

By utilizing Lemma 2, the summations and the fuzzy membership functions will be omitted from inequalities (36). Finally, the results are going to be used for fmincon function which is a nonlinear optimization tool in MATLAB software and finds the minimum of a problem specified by

(37) $$min_{x}f\left(x\right)\;\mathrm{subject}\;\mathrm{to}\;\{\begin{array}{c} c\left(x\right)\leq 0\;\;\;\;\;\;\;\;\;\\ \;ceq\left(x\right)\leq 0\;\;\;\;\\ \begin{array}{c} A\cdot x\leq b\;\;\;\;\;\;\;\;\;\\ \begin{array}{c} Aeq\cdot x=beq\\ \;lb\leq x\leq ub\;\; \end{array} \end{array} \end{array}.$$

The matrix inequalities (36) should be changed to some one-dimensional inequalities, and the optimization problem can be defined as (25) and (26). In addition, from the α and σ found by the optimization problem, the maximum admissible Lipschitz constant and the minimum can be calculated as

(38) $$\gamma^{*}=\frac{1}{∥T_{L}∥∥T_{L}{}^{-1}∥\sqrt{\sigma \varepsilon }}.$$

□

## 4. Simultaneous Fault Reconstruction

In Section 3, an $H_{\infty }$ sliding mode observer is designed in which two discontinuous terms (19) are considered to reconstruct simultaneous faults in the presence of an unknown disturbance based on the measured signals u and y. Along the sliding surface $e_{Y}=\dot{e_{Y}}=0$. Consequently, (22) on the sliding surface changes to

(39) $$\overset{r}{\underset{i=1}{\sum }}{\hat{\mu }}_{i}\left\{\begin{array}{c} T_{0}A_{21.i}e_{1}+T_{0}\phi_{2}+T_{0}{ℳ}_{2i}f_{a}-\\ \nu_{eqa.i}+T_{0}N_{2}f_{s}-\nu_{eqs}+T_{0}D_{2i}d\; \end{array}\right\}=0,$$

where $\nu_{eqa,i}$ and $\nu_{eqs}$ are approximations of the equivalent output error injection terms (17) required to maintain the sliding motion and can be defined as

(40) $$\nu_{eqa.i}=\eta_{a.i}\frac{e_{Y}}{∥e_{Y}∥+\delta_{a}};\;\;\;\;\nu_{eqs}=\eta_{s}\frac{e_{Y}}{∥e_{Y}∥+\delta_{s}},$$

where $\delta_{f}$ and $\delta_{d}$ are small positive constants. Consequently, (40) leads to

(41) $$\begin{array}{c} 0=\overset{r}{\underset{i=1}{\sum }}{\hat{\mu }}_{i}\left\{\begin{array}{c} A_{21.i}e_{1}+\phi_{2}+{ℳ}_{2.i}f_{a}\\ -T_{0}{}^{-1}\nu_{eqa.i}+N_{2}f_{s}-T_{0}{}^{-1}\nu_{eqs}+D_{2.i}d \end{array}\right\} \end{array}.$$

On the other hand, using (8) and (24) can show that the term $\begin{array}{c} A_{21.i}e_{1}+\phi_{2} \end{array}+D_{2.i}d$ is bounded as

(42) $$∥A_{21.i}e_{1}+\phi_{2}+D_{2.i}d∥\;\leq \left(∥A_{21.i}∥+\gamma ∥T_{L}{}^{-1}∥\right)∥e_{1}∥+∥D_{2.i}∥∥d∥\;\leq \left(∥A_{21.i}∥+\gamma ∥T_{L}{}^{-1}∥\right)∥\tilde{e}∥+∥D_{2.i}∥∥d∥\;\leq \epsilon ∥d∥,$$

where $\epsilon =\mu \left(∥A_{21,i}∥+\gamma ∥T_{L}{}^{-1}∥\right)+∥D_{2,i}∥$. Therefore, for small values of ϵ∥d∥, the actuator and sensor faults can be estimated as

(43) $$\hat{f_{a}}={\left(\overset{r}{\underset{k=1}{\sum }}{\hat{\mu }}_{i}\left\{{ℳ}_{2.i}\right\}\right)}^{†}T_{0}{}^{-1}\overset{r}{\underset{k=1}{\sum }}{\hat{\mu }}_{i}\left\{\eta_{a.i}\frac{e_{Y}}{∥e_{Y}∥+\delta_{a}}\right\}$$

(44) $$\hat{f_{s}}=N_{2}^{†}T_{0}{}^{-1}\eta_{s}\frac{e_{Y}}{∥e_{Y}∥+\delta_{s}},$$

where † shows the pseudo-inverse of a matrix.

**Remark** **1.** *The numerical solution of Theorem 1 can be summarized as follows:*

- *Find theorthogonal transfer matrix* $T_{r}\in R^{p\times p}\;$ *by using the QR reduction of matrix* N *and obtain the augmented TS system(4).*
- *Find the changes of coordinates* ${\bar{T}}_{i}$ *and obtain the system matrices in the format* *(12) and* *(13).*
- *Compute the scalars* σ *,* ε *, and* θ *and also the matrices* $L_{i}$ *using the fmincon function in MATLAB software and solving the nonlinear optimization problem* *(25).*
- *Compute the maximized admissible Lipschitz constant as*$\gamma^{*}=\max\left(\gamma \right)=\frac{1}{T_{L}T_{L}{}^{-1}\sqrt{\varepsilon \sigma }}$.
- *Reconstruct the sensor and actuator faults using Equations* *(43) and* *(44).*

## 5. Numerical Example

In this section, a three-state variable continuous stirred tank reactor (CSTR) system is utilized to show the effectiveness of the proposed sliding mode observer in both actuator and sensor faults reconstruction in the presence of an unknown disturbance. To show the performance improvement of the proposed approach, the obtained results are compared to the LMI approach presented in ref. [17].

Consider a well-mixed variable CSTR in which a multi-component chemical reaction A ⇌ B → C is being carried out. The nonlinear dynamics of the CSTR is given by the following model [21],

(45) $$\dot{x}=\left[\begin{array}{ccc} -4 & 0.8796 & 0\\ 3 & -3.6388 & 0\\ 0 & 1.7592 & -1 \end{array}\right]x+\left[\begin{array}{c} 0\\ 1\\ 0 \end{array}\right]u+\left[\begin{array}{c} 0.5x_{2}^{2}\\ -1.5x_{2}^{2}\\ x_{2}^{2} \end{array}\right],$$

where $x={\left[x_{1}\;x_{2}\;x_{3}\right]}^{T},$ and the states represent the concentrations of the species A, B, and C, respectively. To check the advantage of the proposed method, two faults and a disturbance are added to the dynamic (45) as

(46) $$\{\begin{array}{c} \dot{x}=\left[\begin{array}{ccc} -4 & 0.8796+0.5x_{2} & 0\\ 3 & -3.6388-1.5x_{2} & 0\\ 0 & 1.7592+x_{2} & -1 \end{array}\right]x+\left[\begin{array}{c} 0\\ 1\\ 0 \end{array}\right]u\\ +\left[\begin{array}{c} 1\\ 0\\ 0 \end{array}\right]f_{a}+\left[\begin{array}{c} 1\\ 1\\ 1 \end{array}\right]\xi \\ y=\left[\begin{array}{ccc} 0 & 1 & 0\\ 1 & 0 & 0\\ 0 & 0 & 1 \end{array}\right]x+\left[\begin{array}{c} 1\\ 0\\ 0 \end{array}\right]f_{s}\;\;\;\;\;\;\;\;\;\;\;\;\;\;\;\;\;\;\;\;\;\;\;\;\;\;\;\;\;\;\;\;\;\;\; \end{array}.$$

It is supposed that the concentration of B is dimensionless, which means that $x_{2}\in \left[-1\;1\right]$. Consequently, by using TS rules, two membership functions can be defined as

(47) $$h_{1}=\frac{1-x_{2}}{2};\;\;\;h_{2}=\frac{1+x_{2}}{2}.$$

Therefore, the local linear TS matrices can be determined as

(48) $$A_{1}=\left[\begin{array}{ccc} -4 & 0.8796-0.5 & 0\\ 3 & -3.6388+1.5 & 0\\ 0 & 1.7592-1 & -1 \end{array}\right];B_{1}=\left[\begin{array}{c} 0\\ 1\\ 0 \end{array}\right];\;M_{1}=\left[\begin{array}{c} 1\\ 0\\ 0 \end{array}\right];\;D_{1}=\left[\begin{array}{c} 1\\ 1\\ 1 \end{array}\right]A_{2}=\left[\begin{array}{ccc} -4 & 0.8796+0.5 & 0\\ 3 & -3.6388-1.5 & 0\\ 0 & 1.7592+1 & -1 \end{array}\right];B_{2}=\left[\begin{array}{c} 0\\ 1\\ 0 \end{array}\right];M_{2}=\left[\begin{array}{c} 1\\ 0\\ 0 \end{array}\right];\;D_{2}=\left[\begin{array}{c} 1\\ 1\\ 1 \end{array}\right].$$

The TS fuzzy system matrices satisfy all the assumptions; therefore, the TS fuzzy sliding observer (16) can be designed.

**Figure 1:**
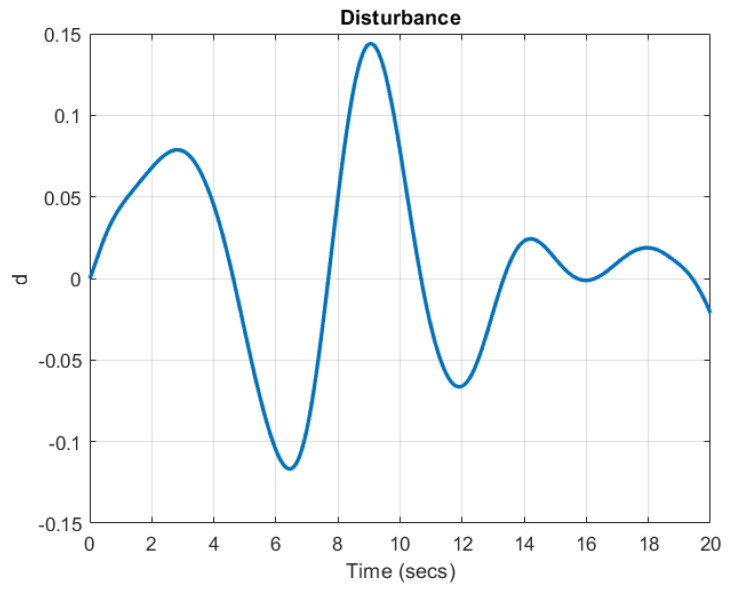
Disturbance d(t).

For simulation, the parameters and input signal are chosen as u=sin(t), $A_{f}=1$, $A_{s}=-5I$, $\eta_{d.i}=\eta_{a}=25$, $\eta_{s}=25$, $\delta_{a}=0.01$ and $\delta_{s}=0.01$. and the initial conditions are chosen as $X_{0}={\left[\begin{array}{cccc} 1 & 1.2 & 1 & 0 \end{array}\right]}^{T}$ and ${\hat{X}}_{0}={\left[\begin{array}{cccc} 1.5 & 2.8 & 0.5 & 0 \end{array}\right]}^{T}$. Moreover, the disturbance is chosen as $d=0.1\sin\left(0.2t\right)x_{3}$ and the shape is shown in Figure 1.

The maximum Lipschitz constant and the minimum $H_{\infty }$ performance gain obtained through fmincon function in MATLAB on Theorem 1 are $\gamma^{*}=0.8358$ and $\vartheta^{*}=0.2982$. The observer matrices are derived as

$$G_{l.1}=[\begin{array}{ccc} 0.4499 & 1 & 0\\ \;3.3912 & 3 & 0\\ 4.8998 & 0 & 0\\ 1.1852 & 0 & 4 \end{array}],\;G_{l.2}=\left[\begin{array}{ccc} 2.4723 & 1 & 0\\ -0.2487 & 3 & 0\\ 8.9447 & 0 & 0\\ 1.7921 & 0 & 4 \end{array}\right],\;G_{n.1}=[\begin{array}{ccc} 0 & 1 & 0\\ \;1.1852 & 0 & 0\\ 1 & 0 & 0\\ 0 & 0 & 1 \end{array}],\;G_{n.2}=\left[\begin{array}{ccc} 0 & 1 & 0\\ 1.7921 & 0 & 0\\ 1 & 0 & 0\\ 0 & 0 & 1 \end{array}\right].$$

**Figure 2:**
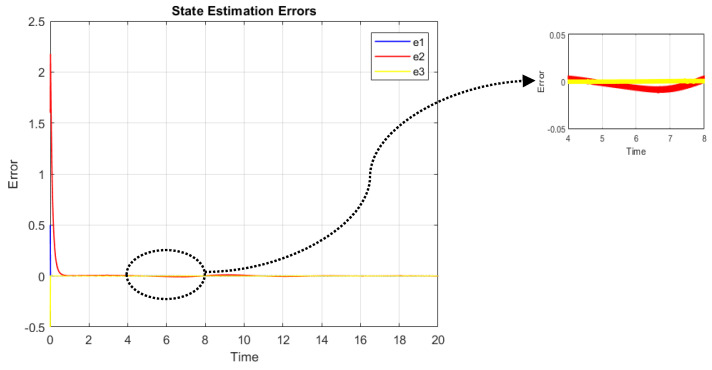
State estimation error in the presence of faults and disturbance.

It should be noted that the initial point for fmincon is chosen based on the results of the related published papers. Figure 2 shows the state estimation error which converges to a neighborhood close to zero due to the unknown disturbance.

**Figure 3:**
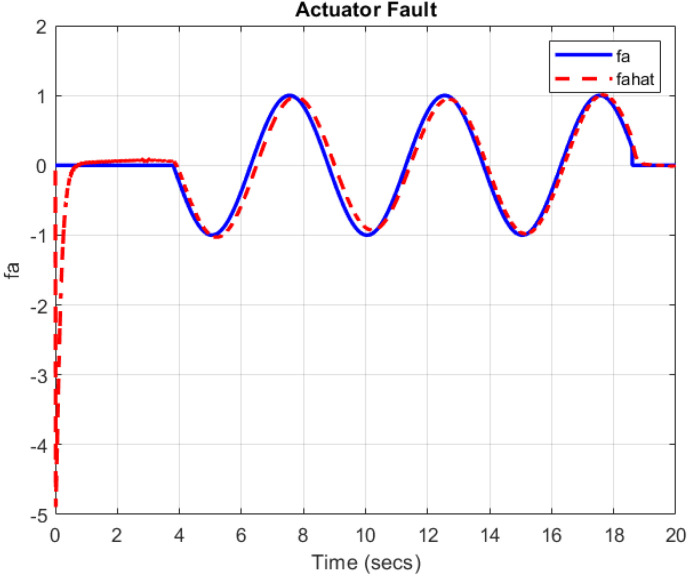
Actuator fault $f_{a}\left(t\right)$ (by blue solid line) and its estimation $\hat{f_{a}}\left(t\right)$ (by red dashed line).

**Figure 4:**
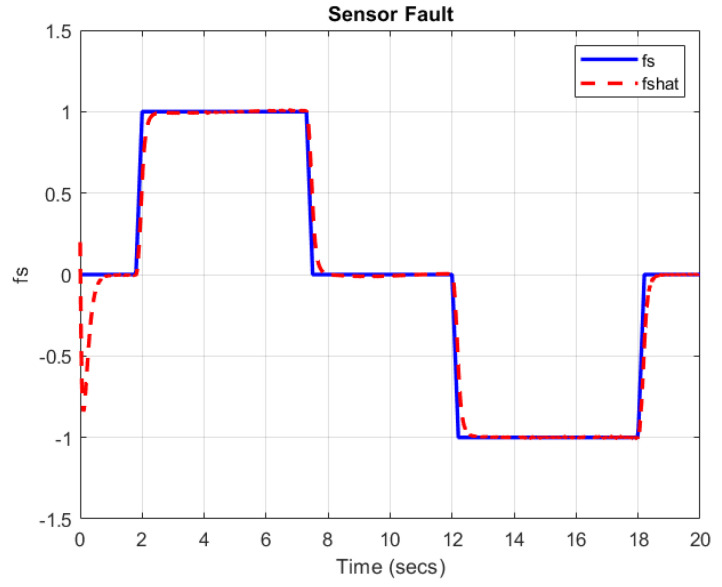
Sensor fault $f_{s}\left(t\right)$ (by blue solid line) and its estimation $\hat{f_{s}}\left(t\right)$ (by red dashed line).

Figure 3 and Figure 4 show that the proposed TS-based SMO is able to reconstruct the simultaneous faults with a small error in the presence of an unknown disturbance.

**Figure 5:**
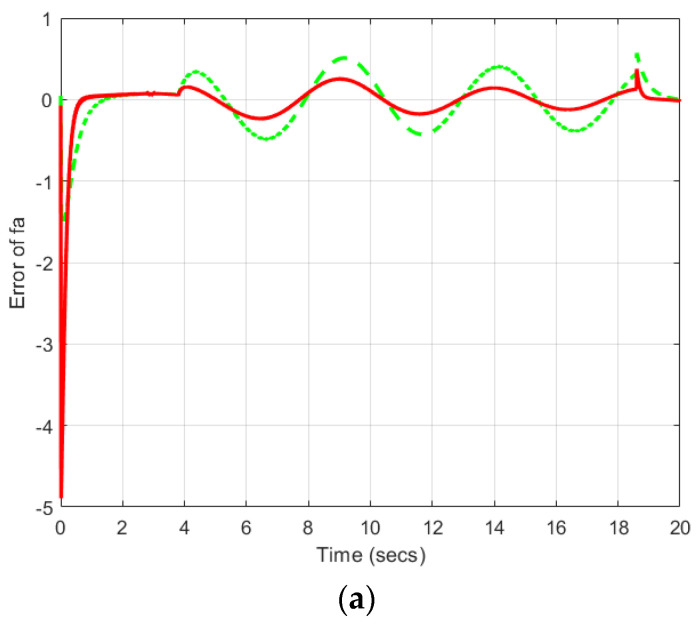

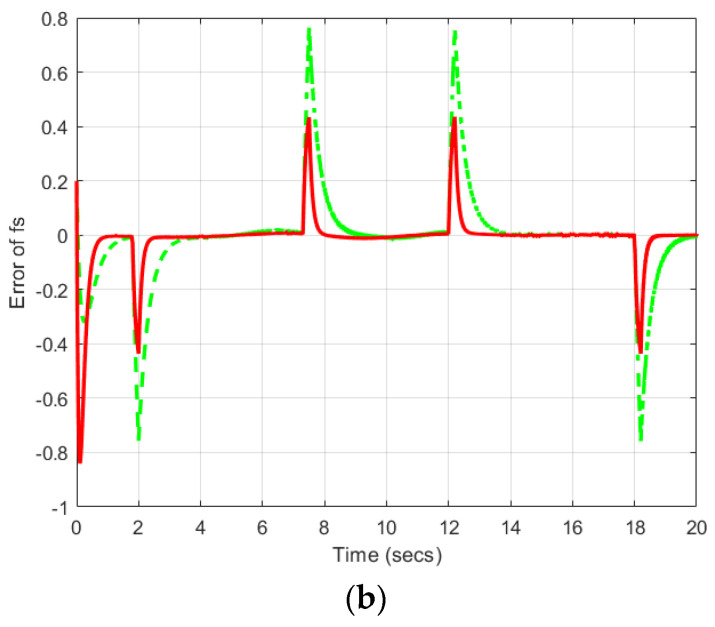
Fault estimation errors (**a**). Actuator fault, (**b**). Sensor fault (the proposed approach by red solid line and ref. [17] by green dashed line).

The proposed approach is compared with another non-quadratic Lyapunov-based approach using linear optimization analysis based on LMIs [17]. Figure 5 describes the fault estimation errors using both approaches.

As can be seen, the proposed nonlinear approach is less conservative and can estimate both actuator and sensor faults with smaller errors. In addition, the proposed approach has less computational burden. In Table 1, a quantitative comparison between the proposed approach and the LMI approach presented in ref. [17] is considered. In this table, the Euclidean and infinity norms of the fault error estimations are compared and the improvements are calculated as

(49) $$Improvement\;\left(\%\right)=\left(\frac{F_{l}-F_{n}}{F_{l}}\right)*100,$$

where $F_{n}$ and $F_{l}$ represent the ∥Error of f∥ using the LMI approach [17] and the nonlinear proposed approach, respectively.

As can be seen in Table 1, the proposed approach improves the fault estimation accuracies by more than 30%.

## 6. Discussion

In this paper, a nonlinear optimization approach for simultaneous actuator and sensor fault reconstruction in nonlinear systems subjected to unknown disturbances was proposed. First, an augmented system with just an actuator fault was created. Then, by using the fuzzy Lyapunov stability analysis and two changes of coordinates, the parameters of a sliding mode observer were designed through a nonlinear optimization problem while maximizing the Lipschitz constant and minimizing the $H_{\infty }$ performance index. The optimization problem was solved by using fmincon in MATLAB as a nonlinear optimization tool. By utilizing the optimum points, both actuator and sensor faults were reconstructed properly. Finally, the simulation results showed a considerable increase in the fault reconstruction accuracy with constraints with smaller dimensions.

## Figures and Tables

**Table 1:** The norm specifications of the fault reconstruction errors for two different approaches.

	$∥ef_{a}∥{}_{2}$	$∥ef_{a}∥{}_{\infty }$	$∥ef_{s}∥{}_{2}$	$∥ef_{s}∥{}_{\infty }$
***Proposed Approach***	23.9858	0.3820	15.3073	0.4376
**[17]**	53.3519	0.5704	33.6076	0.7679
***Improvement (%)***	+55.04	+33.03	+54.45	+43.01

## Data Availability

Not applicable.
